# Establishing a relationship between prolactin and altered fatty acid β-Oxidation via carnitine palmitoyl transferase 1 in breast cancer cells

**DOI:** 10.1186/1471-2407-11-56

**Published:** 2011-02-04

**Authors:** Katja Linher-Melville, Stephanie Zantinge, Toran Sanli, Hertzel Gerstein, Theodoros Tsakiridis, Gurmit Singh

**Affiliations:** 1 Department of Pathology and Molecular Medicine, McMaster University, Hamilton, Ontario, Canada; 2 Department of Medicine, McMaster University, Hamilton, Ontario, Canada; 3 Department of Oncology, McMaster University, Hamilton, Ontario, Canada

## Abstract

**Background:**

Mammary carcinomas have been associated with a high-fat diet, and the rate of breast cancer in overweight post-menopausal women is up to 50% higher than in their normal-weight counterparts. Epidemiological studies suggest that prolactin (PRL) plays a role in the progression of breast cancer. The current study examined breast cancer as a metabolic disease in the context of altered fatty acid catabolism by examining the effect of PRL on carnitine palmitoyl transferase 1 (CPT1), an enzyme that shuttles long-chain fatty acids into the mitochondrial matrix for β-oxidation. The effect of PRL on the adenosine 5'-monophosphate-activated protein kinase (AMPK) energy sensing pathway was also investigated.

**Methods:**

MCF-7 and MDA-MB-231 breast cancer cells and 184B5 normal breast epithelial cells treated with 100 ng/ml of PRL for 24 hr were used as *in vitro *models. Real-time PCR was employed to quantify changes in mRNA levels and Western blotting was carried out to evaluate changes at the protein level. A non-radioactive CPT1 enzyme activity assay was established and siRNA transfections were performed to transiently knock down specific targets in the AMPK pathway.

**Results:**

PRL stimulation increased the expression of CPT1A (liver isoform) at the mRNA and protein levels in both breast cancer cell lines, but not in 184B5 cells. In response to PRL, a 20% increase in CPT1 enzyme activity was observed in MDA-MB-231 cells. PRL treatment resulted in increased phosphorylation of the α catalytic subunit of AMPK at Thr172, as well as phosphorylation of acetyl-CoA carboxylase (ACC) at Ser79. A siRNA against liver kinase B1 (LKB1) reversed these effects in breast cancer cells. PRL partially restored CPT1 activity in breast cancer cells in which CPT1A, LKB1, or AMPKα-1 were knocked down.

**Conclusions:**

PRL enhances fatty acid β-oxidation by stimulating CPT1 expression and/or activity in MCF-7 and MDA-MB-231 breast cancer cells. These PRL-mediated effects are partially dependent on the LKB1-AMPK pathway, although the regulation of CPT1 is also likely to be influenced by other mechanisms. Ultimately, increased CPT1 enzyme activity may contribute to fueling the high energy demands of cancer cells. Targeting metabolic pathways that are governed by PRL, which has already been implicated in the progression of breast cancer, may be of therapeutic benefit.

## Background

Prolactin (PRL) is released from the anterior pituitary gland and is known to play an important role during puberty and during lactation by stimulating the growth and differentiation of breast tissue [1]. A large body of literature supports that PRL promotes cell proliferation, survival, migration/invasion, and angiogenesis (reviewed in [2]). While a growing number of epidemiological studies suggest that PRL contributes to the progression of breast cancer, clinical trials with dopamine agonists (bromocriptine) targeting pituitary-derived PRL in serum failed to block cancer progression [3]. However, it has since been shown that PRL may act as an autocrine/paracrine factor in mammary tissue independent of circulating levels, as it and its receptor (PRLR) are expressed in normal and cancerous breast epithelium [4], and PRL is secreted by cultured breast cancer cells at appreciable levels *in vitro *[5,6]. The existence of a functional autocrine/paracrine loop in the breast is further supported by the finding that breast cancer cell growth and survival in the presence of PRL blocking antibodies and antagonists are abrogated [6,7].

PRL plays a reciprocal role in breast epithelial cells and in adipocytes. During lactation, mammary epithelial cells utilize dietary fat, fatty acids mobilized from surrounding adipose tissue, and newly synthesized lipids to produce milk triacylglycerides, a process that is influenced by both the stage of lactation and the diet [8]. Assessment of murine gene expression profiles revealed that during secretory activation at parturition and during active lactation, genes involved in fatty acid β-oxidation are largely down-regulated while those playing a role in lipogenesis are up-regulated, driving lipid substrates to be utilized for milk fat synthesis [8]. High PRL levels at the onset of lactation and during breast-feeding influence cellular metabolism by favoring lipogenesis (reviewed in [9]). One mechanism by which PRL enhances fatty acid biosynthesis in the milk-producing cells of the bovine mammary gland is via the transcription factor signal transducer and activator of transcription 5 (STAT5), which up-regulates the expression of actyl-CoA carboxylase (ACC), the rate-limiting enzyme of fatty acid biosynthesis [10]. In marked contrast to the changes that occur in mammary epithelial cells during lactation, PRL suppresses lipogenic parameters in cultured human mature adipose tissue [11]. This is evidenced by lower concentrations of malonyl CoA, the product of the first committed step in lipogenesis, as well as suppressed expression of the glucose transporter 4 (GLUT4), which plays a role in insulin-dependent glucose uptake [11]. PRL also suppresses lipogenesis in murine adipocytes via STAT5A, which directly binds to the fatty acid synthase (FASN) promoter and represses its transcriptional activation [12].

When a cell experiences high energy demands or is stressed, the adenosine 5'-monophosphate (AMP)-activated protein kinase (AMPK), a highly conserved heterotrimeric enzyme that gauges cellular energy stores, is activated by phosphorylation of its α subunit at Thr172 [13]. AMPK activation leads to either increased glucose uptake or enhanced fatty acid β-oxidation by mediating the phosphorylation and inactivation of ACC at Ser79 [14]. ACC inactivation leads to decreased levels of malonyl CoA, resulting in a lift in the allosteric inhibition on carnitine palmitoyl transferase 1 (CPT1), a transmembrane enzyme located in the outer mitochondrial membrane [15]. CPT1 represents the rate-limiting step of fatty acid β-oxidation [15,16] and catalyzes the transfer of acyl-CoA from a long-chain acyl-CoA ester to carnitine, forming acylcarnitine, which is then able to enter the mitochondria for β-oxidation [17].

While PRL is an important regulator of fatty acid metabolism in normal human breast epithelial cells and adipose tissue and is locally produced in the breast, its role in mediating β-oxidation via CPT1 in cancer cells has not been explored. We therefore examined whether PRL could have differential effects on fatty acid catabolism in breast cancer cells. Our aims were to investigate its putative metabolic role by evaluating 1) PRL-mediated changes in CPT1 expression in breast cancer cells compared to normal breast epithelial cells at the mRNA and protein levels, 2) changes in CPT1 enzyme activity, and 3) activation of the AMPK pathway via phosphorylation of its α subunit at Thr172 and inactivation of ACC via phosphorylation at Ser79. Gaining a better understanding of how hormones and growth factors differentially affect breast cancer cells by altering energy production and/or utilization compared to normal breast epithelial cells may provide new insights into the development of therapies for the treatment of breast cancer.

## Methods

### Cell Culture

All human cell lines were used in accordance with institutional biosafety guidelines. Cells were obtained from ATCC, cultured in T-75 flasks (Corning), and passaged at least twice post-thaw prior to use in experiments. MCF-7 and MDA-MB-231 were maintained in DMEM, High Glucose (Invitrogen) supplemented with 10% fetal bovine serum (FBS) at 37°C in 5% CO_2_. The 184B5 immortalized normal human breast epithelial cell line was maintained using the MEGM media kit (Clonetics) at 37°C in 5% CO_2_. All cells were passaged no more than 15 times, after which they were discarded.

### Treatment with Recombinant Human PRL

Lyophilized recombinant human PRL (Cedarlane) was reconstituted in sterile water to 100 μg/ml and stored in individual aliquots at -20°C to prevent freeze-thawing. For all experiments other than siRNA transfections, cells were seeded into 6-well plates at 2.5 × 10^5 ^cells per well one day prior to treatment with PRL. The media was changed and PRL was added at final concentrations of 25, 100, and 500 ng/ml. Cells were treated with PRL for 24 hr and harvested for RNA and protein isolation.

### Real Time PCR

Relative mRNA levels were evaluated by real time PCR using cDNA prepared from 184B5, MCF-7, and MDA-MB-231 cells. Reactions were based on an input of 500 ng of total RNA and were carried out using SuperScript III (Invitrogen) following the manufacturer's protocol. Forward (FOR) and reverse (REV) primers used to amplify human *CPT1A *(the liver isoform)*, FASN, LKB1*, and *RPII *were designed based on modifications to existing PCR primer pairs for gene expression detection and quantification listed in PrimerBank, with annealing temperatures of 60°C (http://pga.mgh.harvard.edu/primerbank/index.html). *CPT1A*: FOR 5'-CCTCCAGTTGGCTTATCGTG-3' and REV 5'-TTCTTCGTCTGGCTGGACAT-3', 133 bp; *FASN*: FOR 5'-ACAGCGGGGAATGGGTACT-3'and REV 5'-GACTGGTACAACGAGCGGAT-3', 188 bp; *LKB1*: FOR 5'-GAGCTGATGTCGGTGGGTATG-3'and REV 5'-CACCTTGCCGTAAGAGCCT-3', 144 bp; *RPII-1*: FOR 5'-GGGTGCTGAGTGAGAAGGAC-3' and REV 5'-AGCCATCAAAGGAGATGACG-3', 138 bp; *RPII-2*: FOR 5'-GAAACGGTGGACGTGCTTAT-3' and REV 5'-TCTCCATGCCATACTTGCAC-3', 157 bp. Cycling conditions were as follows: 95°C for 1 min, 40 total cycles of 95°C for 10 sec, 60°C for 25 sec, and melt peak determination (95°C for 15 sec, increasing from 65°C to 95°C with 0.5°C increments for 5 sec each). Parallel reactions were carried out for the *RPII *housekeeping gene to calculate relative mRNA levels by real time PCR using the 2^-[Δ][Δ]Ct ^method [18]. *RPII-1 *was used for *CPT1A *and *LKB1*, and *RPII-2 *was used for *FASN*. The amplification efficiencies were tested for each primer pair, the specificity of the melt curves was assessed, and the integrity of each product was verified by gel electrophoresis.

### Western Blotting

Total cell lysates were harvested by scraping and sonication in 1× lysis buffer (20 mM Tris-Cl pH 7.5, 150 mM NaCl, 1 mM EDTA, 1 mM EGTA, 1% Triton X-100, +Na_2_H_2_P_2_O_7_, +Na_3_VO_4_) supplemented with protease inhibitors (Roche). Protein concentrations of cleared lysates were determined using the Bradford assay. A total of 30 μg of each protein was subjected to SDS-PAGE electrophoresis on 8% polyacrylamide gels. Following transfer onto PVDF membranes and blocking, blots were incubated in primary anti-CPT1A (1:500 dilution, ProteinTech Group, Inc), anti-phospho-ACC at Ser79 (1:1000, Cell Signaling Technology), anti-ACC (total; 1:1000, Cell Signaling Technology), anti-phospho-AMPKα at Thr172 (1:1000, Cell Signaling Technology), anti-AMPKα1 (total; 1:1000, Cell Signaling Technology), or anti-LKB1 (1:1000, Cell Signaling Technology) antibodies followed by incubation with either anti-mouse or anti-rabbit IgG horseradish peroxidase (1:3000, Cell Signaling Technology) as applicable. Signals were detected using the ECL Plus Western Blotting Detection System (Amersham Biosciences) and exposure to film. Stripped membranes were reprobed with primary anti-Actin antibody (1:500, MP) followed by anti-mouse IgG horseradish peroxidase (1:8000). Densitometric analysis was performed using ImageJ software.

### Determination of CPT1 Enzyme Activity

To determine CPT1 enzyme activity, cells were harvested after a 24 hr treatment with 100 ng/ml of human recombinant PRL. Whole cell lysates were prepared as described for Western blotting. A non-radioactive method to measure CPT1 activity in cleared lysates was modified from existing protocols [19-21] and was based on spectrophotometrically measuring the release of CoA-SH from palmitoyl CoA at an absorbance of 412 nm using the general thiol reagent 5,5'-dithio-bis-(2-nitrobenzoic acid) (DTNB). Reaction mixtures containing DTNB and cell lysates were incubated at room temperature for 30 min to eliminate reactive thiol groups and the resulting background absorbance was measured. To start the reaction, palmitoyl-CoA (100 μM final concentration) prepared in double distilled water and L-carnitine solution (5 mM final concentration in 1 M Tris, pH 8.0) were added to the reaction mixtures. Immediately after the addition of substrates, kinetic reads at 30 sec intervals were collected for 90 min by measuring the absorbance at 412 nm. The difference between absorbance readings with and without substrates measured the release of CoA-SH, and values were corrected for total protein. Activity was defined as nmol CoA-SH released/min/mg protein. The protein content of the cell lysates was determined using the Bradford assay.

### siRNA Transfections

Cells were seeded at 7 × 10^4 ^cells per well into 6-well plates and allowed to adhere by culturing at 37°C, 5% CO_2 _for 4 hr. Transient siRNA transfections were carried out using Hiperfect reagent (Qiagen) and siRNAs specifically targeted against CPT1A, LKB1, and AMPKα-1 (Qiagen) following the manufacturer's recommendations, with modifications [22]. The media was changed 24 hr post-transfection, and 48 hr post-transfection, the media was changed with the addition of a final concentration of 100 ng/ml of recombinant human PRL for 24 hr.

### Statistical Analyses

Mean fold changes in mRNA levels were calculated relative to untreated (-PRL) 184B5 cells. Mean fold changes for enzyme activity assays following siRNA transfection were set relative to untreated vehicle. Data represent the mean ± SEM of three independent experiments. Data were either analyzed by t-test or one-way analysis of variance (ANOVA) followed by the Tukey post-test for multiple comparisons to determined statistical differences between groups (denoted by a star or different letters, respectively) using GraphPad Prism analysis software. Results were considered significant at P < 0.05.

## Results

### *CPT1A *mRNA levels are elevated while *FASN *mRNA levels are suppressed in MCF-7 and MDA-MB-231 breast cancer cells

**Figure 1:**
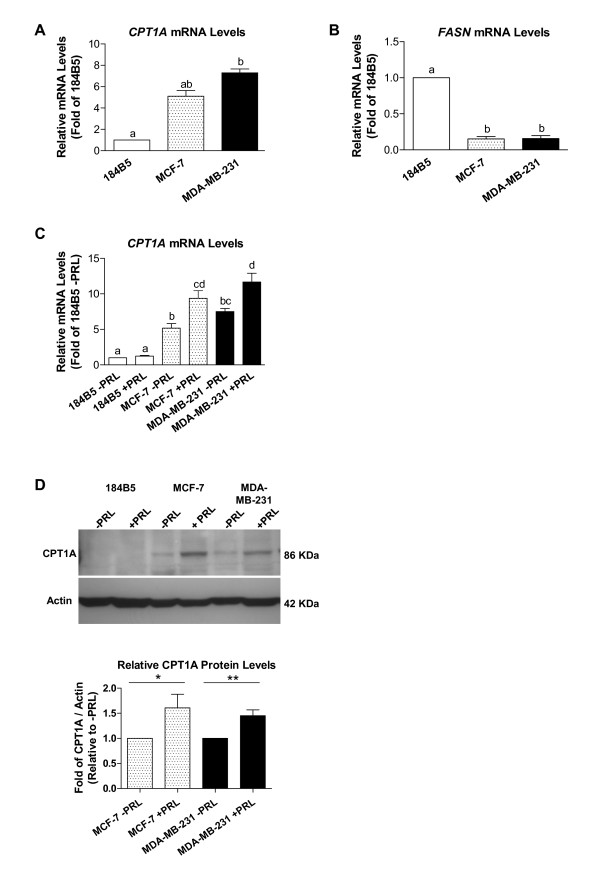
**CPT1A mRNA and protein levels are elevated in breast cancer cells and increase in response to PRL**. Basal mRNA levels were determined by real time PCR, revealing that A) *CPT1A *expression was elevated in both MCF-7 and MDA-MB-231 cells by 5.1 and 7.3 fold, respectively (p < 0.0001), relative to 184B5 cells, while B) *FASN *mRNA levels were over 6-fold lower in both breast cancer cell lines relative to 184B5 cells (p < 0.0001). C) A 100 ng/ml dose of PRL significantly increased *CPT1A *mRNA levels by 1.8- and 1.6-fold in MCF-7 and MDA-MB-231 breast cancer cells, respectively (p < 0.001) relative to untreated cells. In A), B), and C), different letters represent statistical differences between groups. D) A representative Western blot depicting the 86 kDa CPT1A protein (top). Densitometric analysis (bottom) revealed that 100 ng/ml of PRL increased protein levels by 61 and 45% in MCF-7 and MDA-MB-231 cells, respectively. All results represent the mean ± SEM of three independent experiments.

The expression of two genes, *CPT1 *and *FASN*, that play key roles in fatty acid metabolism (lipolytic versus lypogenic) were evaluated at the basal mRNA level in the normal breast epithelial cell line 184B5 and in MCF-7 and MDA-MB-231 breast cancer cells. Relative quantification revealed that *CPT1A *(the liver isoform) mRNA levels were significantly elevated in both MCF-7 and MDA-MB-231 cells compared to 184B5 cells by 5.1 and 7.3 fold, respectively (Figure 1A; p < 0.0001). Furthermore, *FASN *mRNA levels were over 6-fold lower in both breast cancer cell lines compared to 184B5 cells (Figure 1B; 0.15-fold compared to 1-fold, respectively, p < 0.0001).

### PRL significantly increases CPT1A mRNA and protein levels in MCF-7 and MDA-MB-231 breast cancer cells

A 24 hr incubation with 100 ng/ml of recombinant human PRL significantly increased *CPT1A *mRNA levels by 1.8- and 1.6-fold in MCF-7 and MDA-MB-231 breast cancer cells, respectively (Figure 1C; p < 0.0001 and p < 0.001, respectively) compared to untreated controls. In contrast, *CPT1A *mRNA levels did not significantly change in 184B5 cells in response to treatment with the same dose of PRL (Figure 1C). Similar results at the mRNA level were obtained when cells were stimulated with 500 ng/ml of PRL for 24 hr (results not shown). Stimulation with 100 ng/ml of PRL also increased CPT1A protein levels in MCF-7 and MDA-MB-231 cells by approximately 60 and 45% (1 vs. 1.61 ± 0.27-fold, p < 0.05 and 1 vs. 1.45 ± 0.12-fold, p < 0.005, respectively) as determined by densitometric analysis (Figure 1D). Of note, CPT1A could only be detected at the protein level in 184B5 after prolonged exposure.

### PRL significantly increases CPT1 enzyme activity in MDA-MB-231 breast cancer cells

**Figure 2:**
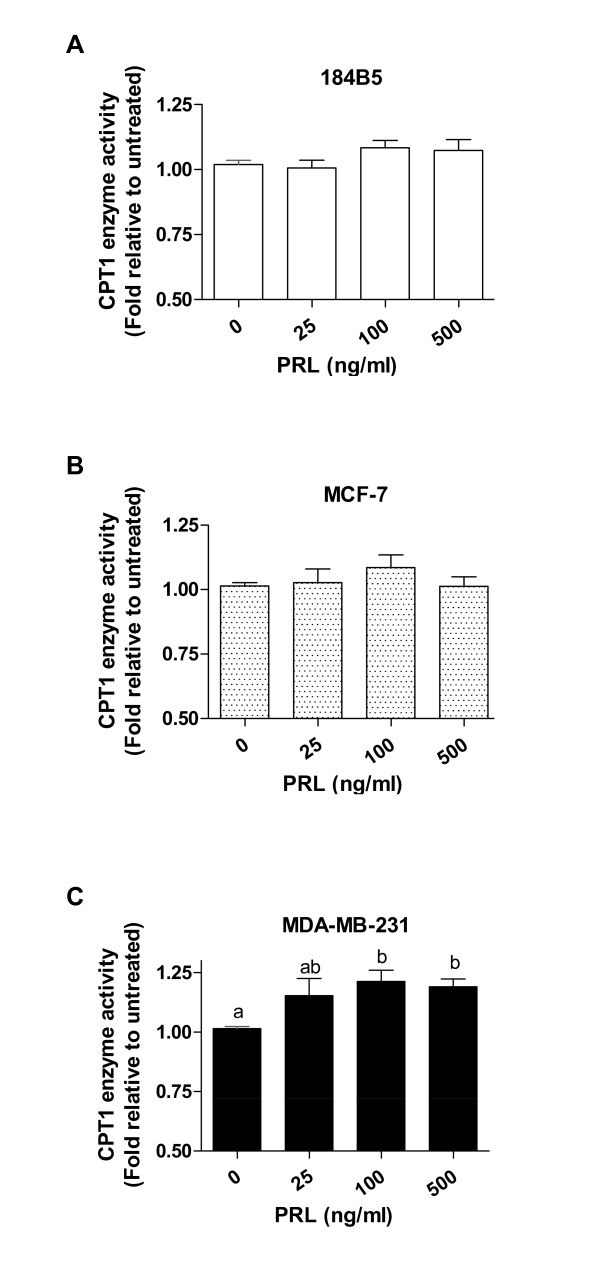
**PRL significantly increases CPT1 enzyme activity in MDA-MB-231 breast cancer cells**. Enzymatic analysis of cell lysates harvested 24 hr following culture in the absence and presence of varying doses of recombinant human PRL (0, 25, 100, and 500 ng/ml). 100 ng/ml of PRL slightly increased CPT1 enzyme activity in A) 184B5 cells by 8.3% and in B) MCF-7 cells by 8.5%. C) CPT1 enzyme activity was significantly enhanced in MDA-MB-231 breast cancer cells treated with 100 and 500 ng/ml of PRL, resulting in 21.2% and 18.8% increases, respectively (p < 0.02). Results are presented as fold of enzyme activity (nmol CoA-SH released/min/mg protein) relative to untreated cells and represent the mean ± SEM of three independent experiments. Different letters represent statistical differences between groups.

To establish whether stimulation with PRL resulted in a functional effect on fatty acid β-oxidation via CPT1, whole cell lysates isolated from cells treated for 24 hr without and with varying doses of recombinant human PRL (0, 25, 100, and 500 ng/ml) in the presence of the substrates palmitoyl CoA and carnitine were analyzed for CPT1 enzyme activity. While not statistically significant, culture of 184B5 and MCF-7 cells with a 100 ng/ml dose of PRL similarly increased CPT1 enzyme activity by 8.3% and 8.5%, respectively (Figure 2A and 2B). In contrast, CPT1 enzyme activity was significantly enhanced in MDA-MB-231 breast cancer cells at both the 100 and 500 ng/ml doses of PRL, resulting in 21.2% and 18.8% increases, respectively, compared to untreated cells (Figure 2C; p < 0.02).

### Stimulation with PRL induces phosphorylation of the AMPKα subunit at Thr172 and phosphorylation of ACC at Ser79

**Figure 3:**
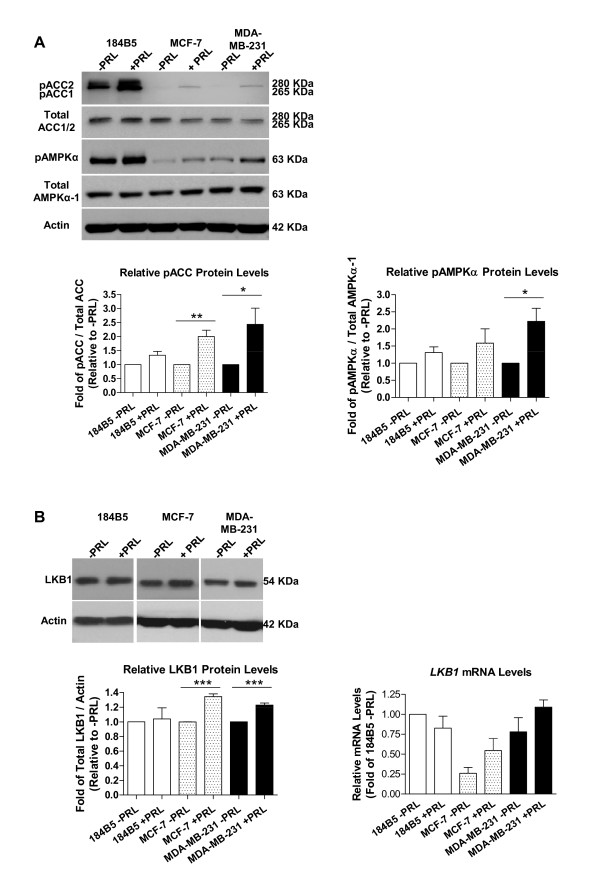
**PRL induces phosphorylation of the AMPKα subunit at Thr172 and increases levels of phosphorylated ACC at Ser79**. Stimulation with 100 ng/ml of PRL increased the levels of A) phosphorylated ACC (pACC) and phosphorylated AMPKα (pAMPKα) in 184B5, MCF-7, and MDA-MB-231 cells as determined by Western blotting. Densitometric analyses revealed significant ~2-fold changes in pACC in MCF-7 and MDA-MB-231 breast cancer cells (p < 0.005 and p < 0.05, respectively), and an increased level of pAMPKα protein in MDA-MB-231 cells (p < 0.02). B) In response to 100 ng/ml of PRL, total LKB1 protein levels were increased by 34 and 23% in MCF-7 and MDA-MB-231 cells, respectively, which was also reflected at the mRNA level. Results represent the mean ± SEM of three independent experiments.

Treatment with 100 ng/ml of recombinant human PRL resulted in a significant increase in the phosphorylation of ACC at Ser79 in MCF-7 and MDA-MB-231 cells (Figure 3A, top panel). Densitometry revealed that in MCF-7 cells, pACC protein levels increased by 2-fold in response to PRL (1 vs. 2.00 ± 0.22-fold, p < 0.005), with a similar result in MDA-MB-231 cells (1 vs. 2.43 ± 0.58-fold, p < 0.05). No significant changes in the phosphorylation status of AMPKα at Thr172 were observed in 184B5 or MCF-7 cells in response to stimulation with the same dose of PRL, while in MDA-MB-231 cells, levels of phosphorylated AMPKα increased by 2-fold (Figure 3A, middle panel; 1 vs. 2.22 ± 0.38-fold, p < 0.02). Liver kinase B1 (LKB1) is one of the key upstream kinases that phosphorylate AMPK at the α subunit [23]. Therefore, changes in total LKB1 expression in response to PRL were also examined at the protein and mRNA levels. The levels of total LKB1 protein were modestly but significantly increased by 34 and 23% following stimulation with PRL in both MCF-7 and MDA-MB-231 breast cancer cells, respectively (Figure 3B; 1-fold vs. 1.34 ± 0.04-fold, p < 0.0001, and 1 vs. 1.23 ± 0.03-fold, p = 0.0002, respectively). In contrast, no significant change was observed in 184B5 cells. Although not statistically significant, a similar trend was reflected at the mRNA level (Figure 3B).

### Transient knock-down of CPT1A, AMPKα-1, and LKB1 leads to decreased CPT1 enzyme activity in MCF-7 and MDA-MB-231 breast cancer cells

**Figure 4:**
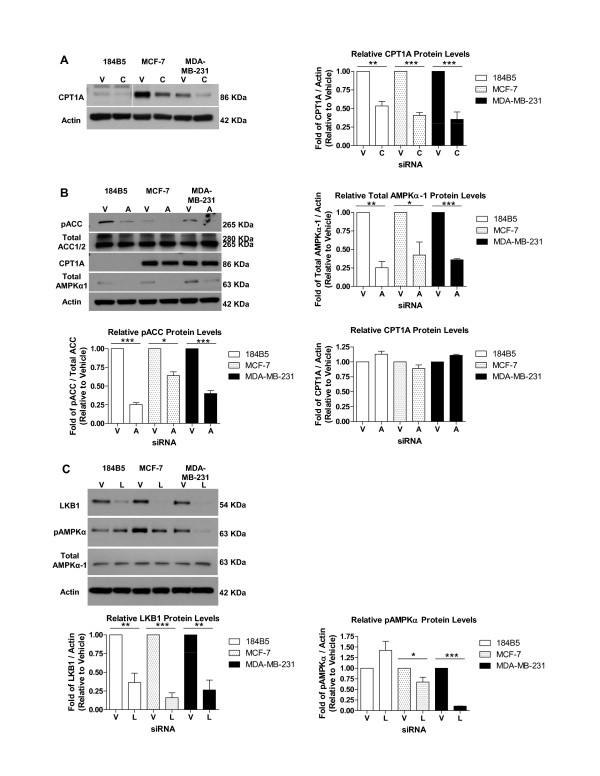
**CPT1A, AMPKα-1, and LKB1 siRNAs effectively knock down expression at the protein level**. The efficiency of the transient siRNA approach was assessed at the protein level by Western blotting coupled with densitometry for A) CPT1A, B) AMPKα-1, and C) LKB1 in transiently transfected 184B5, MCF-7, and MDA-MB-231 cells. Lysates from cells in which B) AMPKα-1 was knocked down were also probed for changes in the levels of phosphorylated ACC (pACC) and CPT1A. Those derived from cells in which C) LKB1 was targeted were further probed for changes in phosphorylated AMPKα (pAMPKα). Vehicle = V, CPT1A siRNA = C, AMPKα siRNA = A, and LKB1 siRNA = L. Results represent the mean ± SEM of three independent experiments.

In order to investigate the effect of PRL on CPT1 enzyme activity via activation of the AMPK pathway, a transient siRNA approach was employed to knock down CPT1A, AMPKα-1, and its primary upstream kinase LKB1. The AMPKα-1 subunit was selected based on our finding that the α-2 subunit was not expressed at the mRNA level in the three cell lines (results not shown). The efficiency of the siRNA approach was assessed at the protein level for CPT1A, AMPKα-1, and LKB1 (Figure 4A, B, and 4C, respectively). As CPT1A protein was expressed at significantly lower levels in 184B5 cells compared to MCF-7 or MDA-MB-231 cells (Figure 4A), the blot verifying knock-down of CPT1A in this cell line was subjected to overnight exposure to allow detection of the band at 86 kDa. In all three cell lines, densitometry verified that CPT1A protein levels were significantly lower in cells treated with siRNA compared to vehicle (Figure 4A; p < 0.003). Knock-down of total AMPKα-1 (Figure 4B; p < 0.03) also corresponded to a significant decrease in the level of phosphorylated ACC in all three cell lines (p < 0.003), although no significant changes in CPT1A protein levels were observed in 184B5, MCF-7, or MDA-MB-231 cells (Figure 4B). Silencing of LKB1 resulted in decreased levels of phosphorylated AMPKα in breast cancer cells (Figure 4C). In MCF-7 cells, LKB1 was knocked down by 94% (p < 0.0001), also correlating with a significant decrease in phosphorylated AMPKα (Figure 4C; p < 0.05). In MDA-MB-231 cells, the down-stream effects of LKB1 siRNA treatment (84% knock-down, p < 0.002) were more dramatic, as the levels of phosphorylated AMPKα were decreased by 90% (Figure 4C; p < 0.0001). While treatment with this specific siRNA did reduce LKB1 protein levels by 64% in 184B5 cells (p < 0.001), no change in the phosphorylation status of AMPKα was observed (Figure 4C).

**Figure 5:**
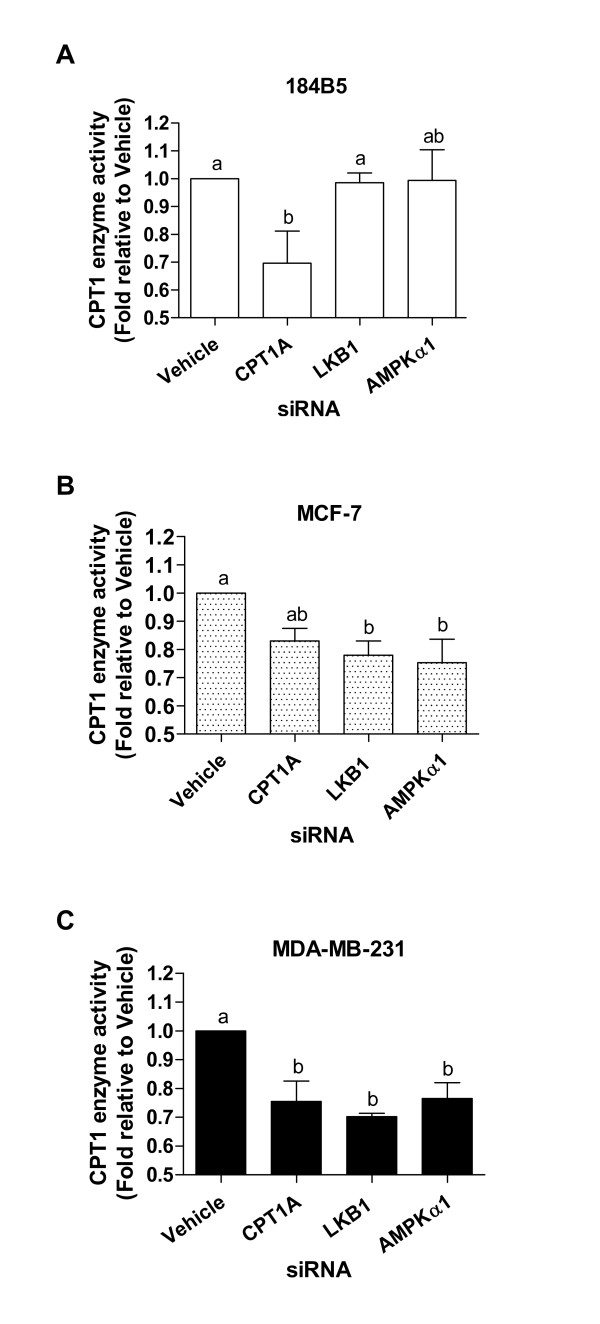
**CPT1A, LKB1, and AMPKα-1 siRNAs decrease CPT1 enzyme activity in MCF-7 and MDA-MB-231 breast cancer cells**. CPT1 enzyme activity assays were performed on cells transiently transfected with siRNAs targeting CPT1A, LKB1, or AMPKα-1. A) In 184B5 cells, treatment with CPT1A siRNA decreased enzyme activity by 30% relative to vehicle (p < 0.05). B) CPT1 enzyme activity was decreased by 17, 22, and 30% relative to vehicle in MCF-7 cells treated with CPT1A, LKB1, and AMPKα-1 siRNAs, respectively (p < 0.05). C) In MDA-MB-231 cells, knock-down of CPT1A resulted in a 24% reduction in enzyme activity relative to vehicle (p < 0.05), and LKB1 and AMPKα-1 siRNAs led to 29 and 23% reductions relative to vehicle, respectively (p < 0.05). Different letters represent statistical differences between groups. Results represent the mean ± SEM of three independent experiments.

CPT1 enzyme activity was significantly decreased by 30% in 184B5 cells treated with CPT1A siRNA compared to vehicle (Figure 5A; 0.70 ± 0.11-fold relative to vehicle, p < 0.05). No changes in enzyme activity were observed using siRNAs against either LKB1 or AMPKα-1 (Figure 5A; 0.99 ± 0.04 and 0.99 ± 0.11-fold relative to vehicle, respectively). As shown in Figure 5B, in MCF-7 cells, CPT1 enzyme activity was decreased by 17, 22, and 25% following transient transfection with CPT1A, LKB1, and AMPKα-1 siRNAs, respectively (0.83 ± 0.04, 0.78 ± 0.05, and 0.75 ± 0.08-fold relative to vehicle, respectively, p < 0.01). Similar decreases were observed in MDA-MB-231 cells, in which treatment with CPT1A siRNA resulted in a 24% reduction in enzyme activity (Figure 5C; 0.76 ± 0.07-fold relative to vehicle, p < 0.001) and treatment with siRNAs targeting LKB1 and AMPKα-1 led to 29 and 23% reductions, respectively (Figure 5C; 0.70 ± 0.01 and 0.77 ± 0.06-fold relative to vehicle, respectively, p < 0.001).

### Decreased CPT1 enzyme activity due to knock-down of CPT1A, LKB1, and AMPKα-1 is partially attenuated by treatment with PRL in breast cancer cells

**Figure 6:**
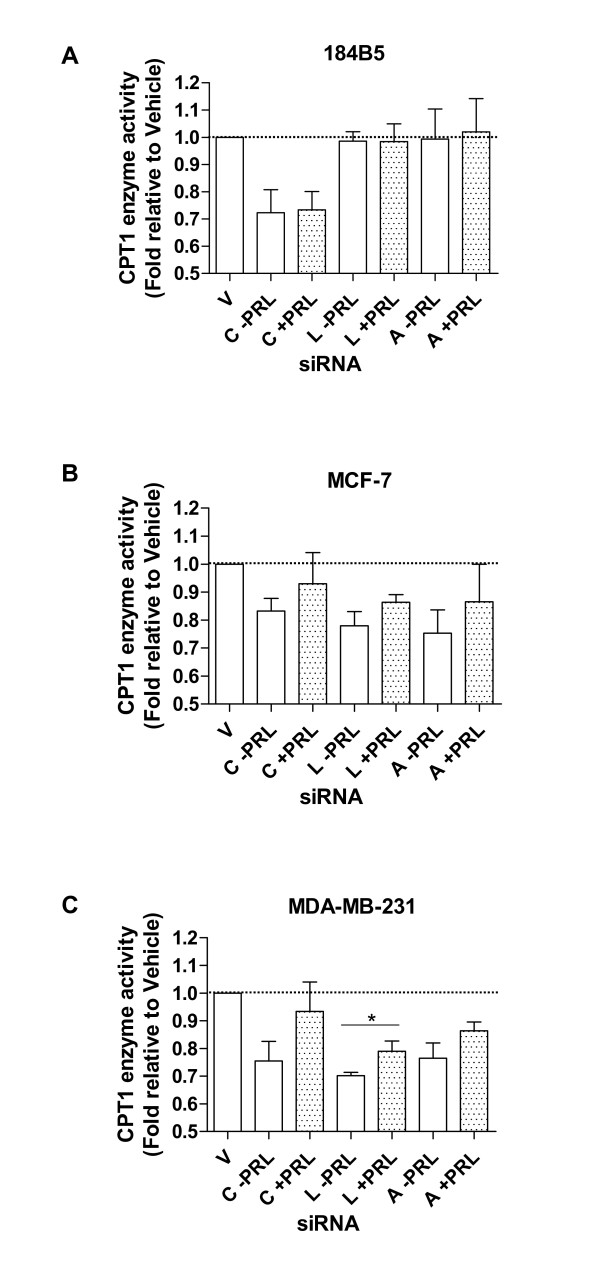
**Decreased CPT1 enzyme activity due to knock-down of CPT1A, LKB1, and AMPKα-1 is partially attenuated by PRL in breast cancer cells**. A) Treatment with 100 ng/ml of PRL for 24 hr had no effect on CPT1 enzyme activity in 184B5 cells transiently transfected with CPT1A siRNA. In both B) MCF-7 and C) MDA-MB-231 breast cancer cells, stimulation of CPT1A siRNA-transfected cells with PRL increased enzyme activity by 10 and 17%, respectively. A PRL-mediated recovery of 8 and 9% was also observed in B) MCF-7 and C) MDA-MB-231 cells, respectively, in which LKB1 was knocked down. In AMPKα1 siRNA-transfected B) MCF-7 and C) MDA-MB-231 cells, PRL increased enzyme activity by 12 and 9%, respectively. The dotted line corresponds to baseline, representing treatment with vehicle only. Vehicle = V, CPT1A siRNA = C, LKB1 siRNA = L, and AMPKα siRNA = A. Results represent the mean ± SEM of three independent experiments.

**Figure 7:**
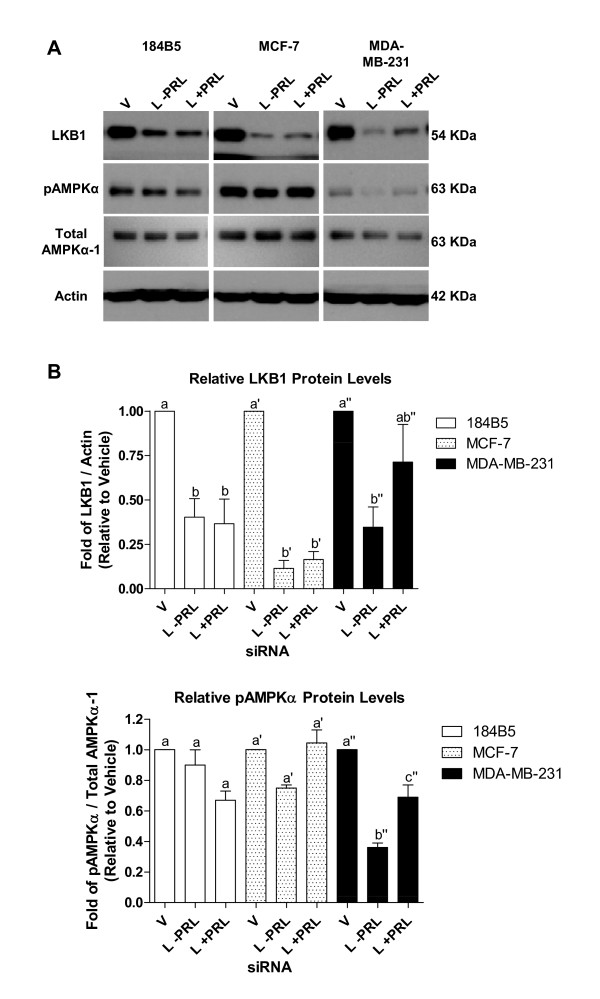
**PRL partially restores total LKB1 and phosphorylated AMPKα protein levels in breast cancer cells in which LKB1 is transiently knocked down**. A) Representative Western blots depicting PRL-mediated changes in total LKB1 protein levels and the corresponding changes in phosphorylated AMPKα (pAMPKα) in 184B5, MCF-7, and MDA-MB-231 cells in which LKB1 was transiently knocked down. B) Densitometry revealed that PRL induced a 2-fold increase in the level of total LKB1 protein in MDA-MB-231 cells. No changes were observed in 184B5 cells that underwent a similar treatment. Increased levels of pAMPKα were also observed in MCF-7 and MDA-MB-231 cells (1.4- and 1.9-fold relative to untreated, respectively) in response to PRL. Different letters represent statistical differences between groups, with ' and " indicate changes within a cell type. Vehicle = V and LKB1 siRNA = L. Results represent the mean ± SEM of three independent experiments.

Treatment of cells transiently transfected with CPT1A siRNA with 100 ng/ml of PRL for 24 hr had no effect on restoring enzyme activity in 184B5 cells (Figure 6A). In marked contrast, in both MCF-7 and MDA-MB-231 breast cancer cells in which CPT1A was knocked down, PRL increased enzyme activity by 10 and 17%, respectively (Figure 6B and 6C), although levels were not restored to baseline (reflected by treatment with vehicle only). In each case, abrogated enzyme activity following treatment with PRL was restored by 60 and 71%, respectively. A similar, although not as dramatic, PRL-mediated recovery was observed in MCF-7 and MDA-MB-231 cells in which LKB1 was knocked down. Treatment with PRL restored CPT1 enzyme activity by 8 and 9% (p < 0.05 for MDA-MB-231 cells), respectively, corresponding with an overall recovery of 36 and 30% (Figure 6B and 6C). In addition, in MCF-7 and MDA-MB-231 cells in which AMPKα-1 was targeted with siRNA, PRL increased enzyme activity by 12 and 9%, respectively (Figure 6B and 6C). This corresponded to an overall recovery toward baseline of 48% and 39%, similar to what was observed for LKB1 in these cells. Coupled with the enzyme activity data, PRL restored total LKB1 protein levels in both LKB1 siRNA-transfected breast cancer cell lines (Figure 7). This effect was more dramatic in MDA-MB-231 cells, reflected by an overall 2-fold increase (Figure 7, top right panel; from 0.35 ± 0.11-fold to 0.71 ± 0.21-fold relative to vehicle only, p < 0.05). In addition, PRL-mediated increases in the level of phosphorylated AMPKα were also more pronounced in MDA-MB-231 cells in which LKB1 was silenced (Figure 7, middle right panel; from 0.36 ± 0.04-fold to 0.69 ± 0.11-fold relative to vehicle only, p < 0.007) compared to MCF-7 cells. This rescue was not evident in 184B5 cells.

## Discussion

Changes in the homeostasis of fatty acid metabolism not only influence the production of cellular energy, but may also have a dramatic impact on an individual's health. Indeed, deregulated lipid metabolism has been linked to the development of obesity, cardiovascular disease, insulin resistance and type 2 diabetes, and cancer [24]. Of clinical relevance, it has recently been shown that enhanced fatty acid catabolism is linked to chemoresistance in leukemia [25], and pharmacologically inhibiting fatty acid β-oxidation sensitizes human leukemia cells to the induction of apoptosis [26]. Despite decades of research, breast cancer remains one of the most common cancers world-wide and a leading cause of cancer death. In the current study, we have examined breast cancer as a metabolic disease, with a focus on lipid metabolism at the molecular level.

A significant body of research has evaluated the lipogenic role of FASN in breast cancer [27-29], and previous work by Mazzarelli et al. (2007) has reported on the expression of CPT1, the rate-limiting enzyme governing long-chain fatty acid catabolism, at the mRNA and protein levels in MCF-7 cells [30]. In addition, it has been suggested that in certain cancer cell types, a metabolic shift may occur in which high rates of glycolysis promote increased fatty acid oxidation (references in [26]). It is clear that altered fat metabolism contributes to cancer. However, a detailed analysis of the expression and activity of the mitochondrial CPT1 shuttle in breast cancer cells compared to normal breast epithelial cells has, to our knowledge, not been performed. While PRL has been linked with the progression of breast cancer by promoting cell proliferation, survival, migration/invasion, and angiogenesis (reviewed in [2]), and is known to differentially affect fatty acid metabolism in breast epithelial cells and adipocytes, its effect on CPT1 in breast cancer cells has also not been investigated. We show here that PRL enhances the expression and/or activity of CPT1 via activation of the AMPK pathway in breast cancer cells.

Two CPT1 isoforms, encoded by the liver (CPT1A) and the muscle/heart (CPT1B) form, are expressed in a range of tissues and give rise to enzymes with different kinetic properties [17]. We examined the expression of CPT1A, which is more widely expressed, and determined that basal mRNA levels were markedly higher in both MCF-7 and MDA-MB-231 breast cancer cells compared to the immortalized normal breast epithelial cell line 184B5. We also investigated the expression of *FASN *as a representative lipogenic gene. FASN catalyzes the synthesis of palmitate from malonyl CoA and acetyl CoA via NADPH and is minimally expressed in most normal human tissues, with the exception of cycling endometrium, the lactating breast, and aggressive subsets of human carcinomas, including certain breast cancers [31,32]. We found that *FASN *mRNA levels were significantly lower in both MCF-7 and MDA-MB-231 cells compared to 184B5 cells, suggesting that lipolytic processes were predominant in these particular breast cancer cell lines.

Treatment with PRL further increased the expression of CPT1A at both the mRNA and protein levels in breast cancer cells, with no changes observed in normal breast epithelial cells. PRL also enhanced CPT1 enzyme activity, inducing a more dramatic increase in MDA-MB-231 cells compared to MCF-7 or 184B5 cells. Furthermore, PRL partially rescued the knock-down of CPT1 enzyme activity induced by siRNA-mediated targeting of CPT1A in breast cancer cells, but not in normal breast epithelial cells. While CPT1 is recognized to be an important regulatory check-point in controlling the rate of fatty acid β-oxidation, increases in CPT1 protein levels may not be sufficient to enhance enzyme activity, given the known allosteric inhibition of CPT1 by malonyl CoA. Relevant to our findings in breast cancer cells, Bruce et al. (2007) have shown that fatty acid β-oxidation may nevertheless be activated by increasing the level of CPT1 protein in both isolated mitochondria and intact skeletal muscle [33]. These observations provide evidence that parameters other than the phosphorylation status of ACC and resulting changes in malonyl CoA levels may contribute to the regulation of fatty acid β-oxidation by restoring the functional content of CPT1 enzyme present in the outer mitochondrial membrane. One possible explanation for the finding that PRL increased CPT1 enzyme activity only in MDA-MB-231 cells and not in MCF-7 cells, despite increasing CPT1 expression in both cell lines, is that estradiol may limit CPT1-mediated fatty acid oxidation. This notion is supported by data derived from studies in both rats and humans. It has been shown that injecting rats with estradiol lowers hepatic CPT1 activity, also increasing the sensitivity of CPT1 to its allosteric inhibitor, malonyl CoA [34]. In women, fatty acid oxidation is reduced by oral estrogen administration [35,36]. Given that MCF-7 cells are estrogen receptor-positive, this presents an interesting point to address in future studies. It is possible that although CPT1 protein levels increase in response to PRL in MCF-7 cells, the enzyme may be more sensitive to allosteric inhibition by malonyl CoA, which could be associated with the effects of estrogen.

Activation of the AMPK pathway depends on the cellular AMP:ATP ratio, which changes during states of high energy expenditure that occur during carcinogenesis and in response to stress stimuli [37-40]. The metabolic effects of AMPK are mediated by several distinct mechanisms, including the phosphorylation/dephosphorylation of down-stream targets [37,38,41]. We have shown that treatment of breast cancer cells, particularly MDA-MB-231 cells, with PRL appreciably increases phosphorylation of the α subunit of AMPK at Thr172 beyond basal levels, which is linked with the phosphorylation and inactivation of one of its downstream effectors, ACC. PRL also imparts a partial recovery of CPT1 enzyme activity in breast cancer cells in which AMPKα-1 is transiently knocked down, indicating that PRL-mediated AMPK activation contributes to the changes in CPT1 enzyme activity reported here. One potential relationship linking cellular metabolism and cancer is the interplay between AMPK and LKB1, a constitutively active serine/threonine kinase (reviewed in [13,24]). From a mechanistic perspective, the LKB1-AMPK pathway is activated in response to metabolic stresses that either inhibit ATP production or accelerate ATP consumption [42], as is the case in cancer cells. LKB1 provides constant basal phosphorylation at Thr172 of the AMPKα subunit, thereby preventing dephosphorylation in response to changes in the AMP:ATP ratio [24]. While LKB1 acts as the primary upstream kinase of AMPKα and serves as a key component of physiological AMPK activation [23], calcium/calmodulin kinase kinase β (CAMKKβ) may also phosphorylate the AMPKα subunit [43], and TGFβ-activated kinase 1 (TAK1) has been associated with the AMPK energy sensing pathway [44]. We found that LKB1 acts as the primary upstream kinase leading to activation of AMPK in breast cancer cells, as CAMKKβ could not be detected by Western blot (results not shown) and was therefore not analyzed further, and knock-down of TAK1 using an siRNA approach did not affect CPT1 enzyme activity or the phosphorylation status of AMPKα (results not shown).

In our model, LKB1 is associated with PRL-mediated changes in CPT1 activity in breast cancer cells. One putative means by which PRL increases functional enzyme activity could be by increasing the expression of total LKB1. This notion is supported by the finding that PRL increases total LKB1 protein levels in breast cancer cells in which LKB1 is transiently knocked down, most notably in MDA-MB-231 cells. Nevertheless, the partial rather than full recovery elicited by PRL suggests that, in addition to changes in fatty acid β-oxidation imparted by LKB1-AMPKα, CPT1 activity is also likely to be influenced by other mechanisms. Of note, while AMPKα and ACC were both phosphorylated in normal breast epithelial cells treated with PRL, no significant changes in CPT1 enzyme activity were observed. This could potentially be related to the sensitivity of these cells to malonyl CoA, which remains to be investigated.

The action of AMPK on lipid metabolism may also be mediated at the level of gene transcription via modulation of transcription factors [45]. For example, AMPK activation suppresses the expression of sterol regulatory element binding transcription factor 1 (SREBP-1), which has been shown to regulate the expression of genes associated with fatty acid and triglyceride biosynthesis, including FASN [46]. AMPK also phosphorylates p300, thereby inhibiting its interaction with nuclear receptors such as peroxisome proliferator-activated receptors (PPARs) and thyroid hormone, retinoic acid, and retinoid X receptors, which in turn may either activate or repress the expression of diverse target genes that play a role in lipid metabolism [47]. The partial recovery of CPT1 enzyme activity in response to PRL in breast cancer cells suggests that AMPK activation may directly affect the expression of genes involved in modulating lipid catabolism, thereby contributing to changes observed at the level of CPT1 in a manner independent of ACC inactivation and a lift in the allosteric inhibition due to changing malonyl CoA levels. Another possibility is that regulation of these putative genes occurs in an LKB1-AMPK-independent manner. Interestingly, PPARα is an important activator of fatty acid catabolism and may be activated not only by AMPK, but also by fatty acids, fibrates, and eicosanoids [48]. Tachibana et al. (2005) have shown that *CPT1A *is a PPAR-responsive gene, and that induction of PPARα in human hepatocytes increases *CPT1A *mRNA levels by 2-fold [49]. Our analysis of the human PPARα promoter region led to the identification of a putative STAT5A/B binding site [TTC(T/C)N(G/A)GAA, where N represents any nucleotide; the site identified by computational analysis in the PPARα promoter is TTCCAAGAA]. As PRL mediates its effects via, among others, JAK2/STAT5 signaling in breast cancer cells [50], this could provide a means by which PRL could directly activate PPARα transcription independent from LKB1 and AMPK. It will be of considerable interest to evaluate the contribution of PPARα on changes in CPT1A expression and activity in breast cancer cells in response to PRL in future studies. Importantly, PRL does not restore CPT1 enzyme activity in normal breast epithelial cells in which CPT1A, LKB1, or AMPKα-1 are knocked down, suggesting that distinct pathways are activated in normal and malignant cells.

## Conclusions

In the current study, we have shown that PRL enhances both the expression and activity of CPT1, the rate-limiting enzyme of mitochondrial long-chain fatty acid β-oxidation, in breast cancer cells *in vitro*. Our results reflect what occurs in mature human adipose tissue, and this altered regulation of lipid metabolism in breast cancer cells compared to normal breast epithelial cells in response to PRL could be a means by which nutrients such as lipids are utilized to fuel the high energy demands of cancer cells. Otto Warburg first identified that metabolism is altered in cancer cells, manifested by an increase in glucose uptake and preferential ATP production via glycolysis [51]. It has since been suggested that in certain cancers, a high rate of glycolysis, as well as glutamine metabolism, may actually support the more efficient oxidation of fatty acids in the mitochondria (references in [26]). Of functional relevance, inhibition of CPT1-mediated fatty acid oxidation has been shown to enhance the production of ceramide and to increase apoptosis [52], and application of a pharmacologic inhibitor of CPT1, etomoxir, results in cytotoxicity [53]. These findings suggest that inhibition of fatty acid oxidation may lead to an overall decrease in cancer cell survival, while an increase in CPT1 activity, such as the PRL-mediated response reported in the current investigation, may provide a supportive environment for breast cancer cells.

**Figure 8:**
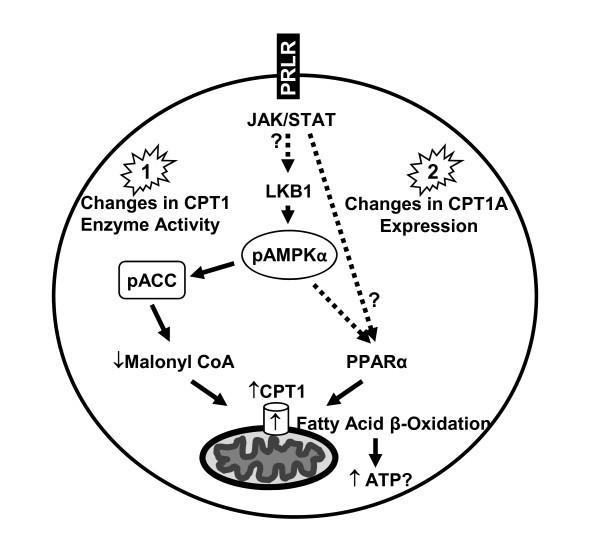
**Proposed mechanism by which PRL may affect CPT1 enzyme activity in breast cancer cells**. PRL signaling may alter CPT1 enzyme activity in two distinct manners, with potential cross-talk. In 1), PRL mediates its effect through LKB1, resulting in activation of the AMPK pathway via phosphorylation of AMPKα at Thr172 (pAMPKα) and inactivation of ACC (pACC), culminating in increased CPT1 enzyme activity. In 2), PRL either directly, or indirectly through the activation of AMPK, induces PPARα to increase CPT1A expression, resulting in changes at both the mRNA and protein levels, thereby increasing CPT1 enzyme activity. Dashed arrows and ?s indicate components of the pathway(s) that remain to be conclusively established.

We propose a mechanism by which PRL elicits its effect on CPT1A in representative breast cancer cells (Figure 8). PRL signaling may positively influence CPT1 enzyme activity by first eliciting an increase in total LKB1 protein levels, in turn leading to increased LKB1-mediated activation of the AMPK pathway via phosphorylation of AMPKα at Thr172 and inactivation of ACC. Another possible scenario is that PRL, either directly or indirectly through the activation of AMPK, stimulates PPARα to increase CPT1A expression at the mRNA and protein levels, thereby potentially increasing CPT1 enzyme activity. To our knowledge, this is the first study demonstrating that LKB1 plays a role in activating the AMPK pathway and thereby CPT1 activity in response to PRL stimulation in breast cancer cells. Ultimately, targeting metabolic pathways that are governed by PRL, which has already been implicated in the progression of breast cancer, may be of therapeutic benefit.

## Abbreviations

ACC: acetyl-CoA carboxylase; AMP/ATP: adenosine monophosphate/adenosine triphosphate; AMPK: 5'-monophosphate-activated protein kinase; CAMKKβ: calcium/calmodulin kinase kinase β; CPT1: carnitine palmitoyl transferase 1; FASN: fatty acid synthase; LKB1: liver kinase B1; PPARα: peroxisome proliferator-activated receptor α; PRL: prolactin; PRLR: prolactin receptor; STAT5: signal transducer and activator of transcription 5; TAK1: TGFβ activating kinase 1

## Competing interests

The authors declare that they have no competing interests.

## Authors' contributions

KL conceived and designed the study, conducted all experiments, performed statistical analyses, and drafted the manuscript. SZ assisted with cell culture and participated in siRNA experiments. TS contributed to critically reviewing the experimental design and the manuscript. TT critically reviewed the manuscript. HG was a co-applicant in providing funding for the study and critically reviewed the manuscript, and GS provided funding and critically reviewed the manuscript. All authors have read and approved the final manuscript.

## Pre-publication history

The pre-publication history for this paper can be accessed here:

http://www.biomedcentral.com/1471-2407/11/56/prepub

## References

[B1] Binart N Ormandy CJ Kelly PA Mammary gland development and the prolactin receptor Adv Exp Med Biol 2000 480 85 92 full_text 10959413 10.1007/0-306-46832-8_10

[B2] Clevenger CV Furth PA Hankinson SE Schuler LA The role of prolactin in mammary carcinoma Endocr Rev 2003 24 1 1 27 10.1210/er.2001-0036 12588805 PMC1698952

[B3] Anderson E Ferguson JE Morten H Shalet SM Robinson EL Howell A Serum immunoreactive and bioactive lactogenic hormones in advanced breast cancer patients treated with bromocriptine and octreotide Eur J Cancer 1993 29A 2 209 217 10.1016/0959-8049(93)90178-I 8422285

[B4] Fields K Kulig E Lloyd RV Detection of prolactin messenger RNA in mammary and other normal and neoplastic tissues by polymerase chain reaction Lab Invest 1993 68 3 354 360 8450651

[B5] Clevenger CV Chang WP Ngo W Pasha TL Montone KT Tomaszewski JE Expression of prolactin and prolactin receptor in human breast carcinoma. Evidence for an autocrine/paracrine loop Am J Pathol 1995 146 3 695 705 7534043 PMC1869171

[B6] Ginsburg E Vonderhaar BK Prolactin synthesis and secretion by human breast cancer cells Cancer Res 1995 55 12 2591 2595 7780973

[B7] Ramamoorthy P Sticca R Wagner TE Chen WY In vitro studies of a prolactin antagonist, hPRL-G129R in human breast cancer cells Int J Oncol 2001 18 1 25 32 11115535 10.3892/ijo.18.1.25

[B8] Rudolph MC Neville MC Anderson SM Lipid synthesis in lactation: diet and the fatty acid switch J Mammary Gland Biol Neoplasia 2007 12 4 269 281 10.1007/s10911-007-9061-5 18027074

[B9] Anderson SM Rudolph MC McManaman JL Neville MC Key stages in mammary gland development. Secretory activation in the mammary gland: it's not just about milk protein synthesis! Breast Cancer Res 2007 9 1 204 10.1186/bcr1653 17338830 PMC1851396

[B10] Mao J Molenaar AJ Wheeler TT Seyfert HM STAT5 binding contributes to lactational stimulation of promoter III expressing the bovine acetyl-CoA carboxylase alpha-encoding gene in the mammary gland J Mol Endocrinol 2002 29 1 73 88 10.1677/jme.0.0290073 12200230

[B11] Nilsson LA Roepstorff C Kiens B Billig H Ling C Prolactin suppresses malonyl-CoA concentration in human adipose tissue Horm Metab Res 2009 41 10 747 751 10.1055/s-0029-1224181 19551610

[B12] Hogan JC Stephens JM The regulation of fatty acid synthase by STAT5A Diabetes 2005 54 7 1968 1975 10.2337/diabetes.54.7.1968 15983196

[B13] Motoshima H Goldstein BJ Igata M Araki E AMPK and cell proliferation--AMPK as a therapeutic target for atherosclerosis and cancer J Physiol 2006 574 Pt 1 63 71 10.1113/jphysiol.2006.108324 16613876 PMC1817805

[B14] Ha J Daniel S Broyles SS Kim KH Critical phosphorylation sites for acetyl-CoA carboxylase activity J Biol Chem 1994 269 35 22162 22168 7915280

[B15] Murthy MS Pande SV Some differences in the properties of carnitine palmitoyltransferase activities of the mitochondrial outer and inner membranes Biochem J 1987 248 3 727 733 3435481 10.1042/bj2480727 PMC1148610

[B16] Murthy MS Pande SV Malonyl-CoA binding site and the overt carnitine palmitoyltransferase activity reside on the opposite sides of the outer mitochondrial membrane Proc Natl Acad Sci USA 1987 84 2 378 382 10.1073/pnas.84.2.378 3540964 PMC304210

[B17] McGarry JD Brown NF The mitochondrial carnitine palmitoyltransferase system. From concept to molecular analysis Eur J Biochem 1997 244 1 1 14 10.1111/j.1432-1033.1997.00001.x 9063439

[B18] Livak KJ Schmittgen TD Analysis of relative gene expression data using real-time quantitative PCR and the 2(-Delta Delta C(T)) Method Methods 2001 25 4 402 408 10.1006/meth.2001.1262 11846609

[B19] Bieber LL Abraham T Helmrath T A rapid spectrophotometric assay for carnitine palmitoyltransferase Anal Biochem 1972 50 2 509 518 10.1016/0003-2697(72)90061-9 4630394

[B20] Karlic H Lohninger S Koeck T Lohninger A Dietary l-carnitine stimulates carnitine acyltransferases in the liver of aged rats J Histochem Cytochem 2002 50 2 205 212 11799139 10.1177/002215540205000208

[B21] Shin ES Cho SY Lee EH Lee SJ Chang IS Lee TR Positive regulation of hepatic carnitine palmitoyl transferase 1A (CPT1A) activities by soy isoflavones and L-carnitine Eur J Nutr 2006 45 3 159 164 10.1007/s00394-005-0576-5 16362726

[B22] Sanli T Rashid A Liu C Harding S Bristow RG Cutz JC Singh G Wright J Tsakiridis T Ionizing radiation activates AMP-activated kinase (AMPK): a target for radiosensitization of human cancer cells Int J Radiat Oncol Biol Phys 78 1 221 229 20615625 10.1016/j.ijrobp.2010.03.005

[B23] Hawley SA Boudeau J Reid JL Mustard KJ Udd L Makela TP Alessi DR Hardie DG Complexes between the LKB1 tumor suppressor, STRAD alpha/beta and MO25 alpha/beta are upstream kinases in the AMP-activated protein kinase cascade J Biol 2003 2 4 28 10.1186/1475-4924-2-28 14511394 PMC333410

[B24] Fogarty S Hardie DG Development of protein kinase activators: AMPK as a target in metabolic disorders and cancer Biochim Biophys Acta 1804 3 581 591 19778642 10.1016/j.bbapap.2009.09.012

[B25] Harper ME Antoniou A Villalobos-Menuey E Russo A Trauger R Vendemelio M George A Bartholomew R Carlo D Shaikh A Characterization of a novel metabolic strategy used by drug-resistant tumor cells Faseb J 2002 16 12 1550 1557 10.1096/fj.02-0541com 12374777

[B26] Samudio I Harmancey R Fiegl M Kantarjian H Konopleva M Korchin B Kaluarachchi K Bornmann W Duvvuri S Taegtmeyer H Pharmacologic inhibition of fatty acid oxidation sensitizes human leukemia cells to apoptosis induction J Clin Invest 120 1 142 156 10.1172/JCI38942 20038799 PMC2799198

[B27] Milgraum LZ Witters LA Pasternack GR Kuhajda FP Enzymes of the fatty acid synthesis pathway are highly expressed in in situ breast carcinoma Clin Cancer Res 1997 3 11 2115 2120 9815604

[B28] Pizer ES Jackisch C Wood FD Pasternack GR Davidson NE Kuhajda FP Inhibition of fatty acid synthesis induces programmed cell death in human breast cancer cells Cancer Res 1996 56 12 2745 2747 8665507

[B29] Puig T Vazquez-Martin A Relat J Petriz J Menendez JA Porta R Casals G Marrero PF Haro D Brunet J Fatty acid metabolism in breast cancer cells: differential inhibitory effects of epigallocatechin gallate (EGCG) and C75 Breast Cancer Res Treat 2008 109 3 471 479 10.1007/s10549-007-9678-5 17902053

[B30] Mazzarelli P Pucci S Bonanno E Sesti F Calvani M Spagnoli LG Carnitine palmitoyltransferase I in human carcinomas: a novel role in histone deacetylation? Cancer Biol Ther 2007 6 10 1606 1613 10.4161/cbt.6.10.4742 18253084

[B31] Kuhajda FP Fatty-acid synthase and human cancer: new perspectives on its role in tumor biology Nutrition 2000 16 3 202 208 10.1016/S0899-9007(99)00266-X 10705076

[B32] Baron A Migita T Tang D Loda M Fatty acid synthase: a metabolic oncogene in prostate cancer? J Cell Biochem 2004 91 1 47 53 10.1002/jcb.10708 14689581

[B33] Bruce CR Brolin C Turner N Cleasby ME van der Leij FR Cooney GJ Kraegen EW Overexpression of carnitine palmitoyltransferase I in skeletal muscle in vivo increases fatty acid oxidation and reduces triacylglycerol esterification Am J Physiol Endocrinol Metab 2007 292 4 E1231 1237 10.1152/ajpendo.00561.2006 17179390

[B34] Weinstein I Cook GA Heimberg M Regulation by oestrogen of carnitine palmitoyltransferase in hepatic mitochondria Biochem J 1986 237 2 593 596 3800903 10.1042/bj2370593 PMC1147025

[B35] O'Sullivan AJ Crampton LJ Freund J Ho KK The route of estrogen replacement therapy confers divergent effects on substrate oxidation and body composition in postmenopausal women J Clin Invest 1998 102 5 1035 1040 9727072 10.1172/JCI2773 PMC508969

[B36] O'Sullivan AJ Hoffman DM Ho KK Estrogen, lipid oxidation, and body fat N Engl J Med 1995 333 10 669 670 7637741 10.1056/NEJM199509073331018

[B37] Hardie DG Scott JW Pan DA Hudson ER Management of cellular energy by the AMP-activated protein kinase system FEBS Lett 2003 546 1 113 120 10.1016/S0014-5793(03)00560-X 12829246

[B38] Kemp BE Stapleton D Campbell DJ Chen ZP Murthy S Walter M Gupta A Adams JJ Katsis F van Denderen B AMP-activated protein kinase, super metabolic regulator Biochem Soc Trans 2003 31 Pt 1 162 168 12546677 10.1042/bst0310162

[B39] Hardie DG Regulation of fatty acid and cholesterol metabolism by the AMP-activated protein kinase Biochim Biophys Acta 1992 1123 3 231 238 1536860 10.1016/0005-2760(92)90001-c

[B40] Park H Kaushik VK Constant S Prentki M Przybytkowski E Ruderman NB Saha AK Coordinate regulation of malonyl-CoA decarboxylase, sn-glycerol-3-phosphate acyltransferase, and acetyl-CoA carboxylase by AMP-activated protein kinase in rat tissues in response to exercise J Biol Chem 2002 277 36 32571 32577 10.1074/jbc.M201692200 12065578

[B41] Ferre P Azzout-Marniche D Foufelle F AMP-activated protein kinase and hepatic genes involved in glucose metabolism Biochem Soc Trans 2003 31 Pt 1 220 223 12546689 10.1042/bst0310220

[B42] Hardie DG Hawley SA Scott JW AMP-activated protein kinase--development of the energy sensor concept J Physiol 2006 574 Pt 1 7 15 10.1113/jphysiol.2006.108944 16644800 PMC1817788

[B43] Hawley SA Pan DA Mustard KJ Ross L Bain J Edelman AM Frenguelli BG Hardie DG Calmodulin-dependent protein kinase kinase-beta is an alternative upstream kinase for AMP-activated protein kinase Cell Metab 2005 2 1 9 19 10.1016/j.cmet.2005.05.009 16054095

[B44] Xie M Zhang D Dyck JR Li Y Zhang H Morishima M Mann DL Taffet GE Baldini A Khoury DS A pivotal role for endogenous TGF-beta-activated kinase-1 in the LKB1/AMP-activated protein kinase energy-sensor pathway Proc Natl Acad Sci USA 2006 103 46 17378 17383 10.1073/pnas.0604708103 17085580 PMC1859937

[B45] Woods A Azzout-Marniche D Foretz M Stein SC Lemarchand P Ferre P Foufelle F Carling D Characterization of the role of AMP-activated protein kinase in the regulation of glucose-activated gene expression using constitutively active and dominant negative forms of the kinase Mol Cell Biol 2000 20 18 6704 6711 10.1128/MCB.20.18.6704-6711.2000 10958668 PMC86183

[B46] Zhou G Myers R Li Y Chen Y Shen X Fenyk-Melody J Wu M Ventre J Doebber T Fujii N Role of AMP-activated protein kinase in mechanism of metformin action J Clin Invest 2001 108 8 1167 1174 11602624 10.1172/JCI13505 PMC209533

[B47] Yang W Hong YH Shen XQ Frankowski C Camp HS Leff T Regulation of transcription by AMP-activated protein kinase: phosphorylation of p300 blocks its interaction with nuclear receptors J Biol Chem 2001 276 42 38341 38344 10.1074/jbc.C100316200 11518699

[B48] Yoon M The role of PPARalpha in lipid metabolism and obesity: focusing on the effects of estrogen on PPARalpha actions Pharmacol Res 2009 60 3 151 159 10.1016/j.phrs.2009.02.004 19646654

[B49] Tachibana K Kobayashi Y Tanaka T Tagami M Sugiyama A Katayama T Ueda C Yamasaki D Ishimoto K Sumitomo M Gene expression profiling of potential peroxisome proliferator-activated receptor (PPAR) target genes in human hepatoblastoma cell lines inducibly expressing different PPAR isoforms Nucl Recept 2005 3 3 10.1186/1478-1336-3-3 16197558 PMC1262764

[B50] Carver KC Arendt LM Schuler LA Complex prolactin crosstalk in breast cancer: new therapeutic implications Mol Cell Endocrinol 2009 307 1-2 1 7 10.1016/j.mce.2009.03.014 19524120 PMC3190192

[B51] Warburg O On the origin of cancer cells Science 1956 123 3191 309 314 10.1126/science.123.3191.309 13298683

[B52] Paumen MB Ishida Y Muramatsu M Yamamoto M Honjo T Inhibition of carnitine palmitoyltransferase I augments sphingolipid synthesis and palmitate-induced apoptosis J Biol Chem 1997 272 6 3324 3329 10.1074/jbc.272.6.3324 9013572

[B53] Hernlund E Ihrlund LS Khan O Ates YO Linder S Panaretakis T Shoshan MC Potentiation of chemotherapeutic drugs by energy metabolism inhibitors 2-deoxyglucose and etomoxir Int J Cancer 2008 123 2 476 483 10.1002/ijc.23525 18452174

